# Enhanced Photoacoustic Visualisation of Clinical Needles by Combining Interstitial and Extracorporeal Illumination of Elastomeric Nanocomposite Coatings

**DOI:** 10.3390/s22176417

**Published:** 2022-08-25

**Authors:** Mengjie Shi, Semyon Bodian, Simeon J. West, Sanjayan Sathasivam, Ross J. Gordon, Paul Collier, Tom Vercauteren, Adrien E. Desjardins, Sacha Noimark, Wenfeng Xia

**Affiliations:** 1 School of Biomedical Engineering and Imaging Sciences, King’s College London, London SE1 7EH, UK; 2 Department of Medical Physics and Biomedical Engineering, University College London, London WC1E 6BT, UK; 3 Wellcome/EPSRC Centre for Interventional and Surgical Sciences, University College London, London W1W 7TY, UK; 4 Department of Anaesthesia, University College Hospital, London NW1 2BU, UK; 5 Department of Chemistry, University College London, London WC1H 0AJ, UK or; 6 School of Engineering, London South Bank University, London SE1 0AA, UK; 7 Johnson Matthey Technology Centre, Reading RG4 9NH, UK

**Keywords:** photoacoustic imaging, needle visualisation, nanocomposite coatings, minimally invasive surgeries

## Abstract

Ultrasound (US) image guidance is widely used for minimally invasive procedures, but the invasive medical devices (such as metallic needles), especially their tips, can be poorly visualised in US images, leading to significant complications. Photoacoustic (PA) imaging is promising for visualising invasive devices and peripheral tissue targets. Light-emitting diodes (LEDs) acting as PA excitation sources facilitate the clinical translation of PA imaging, but the image quality is degraded due to the low pulse energy leading to insufficient contrast with needles at deep locations. In this paper, photoacoustic visualisation of clinical needles was enhanced by elastomeric nanocomposite coatings with superficial and interstitial illumination. Candle soot nanoparticle-polydimethylsiloxane (CSNP-PDMS) composites with high optical absorption and large thermal expansion coefficients were applied onto the needle exterior and the end-face of an optical fibre placed in the needle lumen. The excitation light was delivered at the surface by LED arrays and through the embedded optical fibre by a pulsed diode laser to improve the visibility of the needle tip. The performance was validated using an ex-vivo tissue model. An LED-based PA/US imaging system was used for imaging the needle out-of-plane and in-plane insertions over approach angles of 20 deg to 55 deg. The CSNP-PDMS composite conferred substantial visual enhancements on both the needle shaft and the tip, with an average of 1.7- and 1.6-fold improvements in signal-to-noise ratios (SNRs), respectively. With the extended light field involving extracorporeal and interstitial illumination and the highly absorbing coatings, enhanced visualisation of the needle shaft and needle tip was achieved with PA imaging, which could be helpful in current US-guided minimally invasive surgeries.

## 1. Introduction

Accurate tracking of invasive devices within the body is of critical importance during surgical interventions such as nerve blocks [1], tumour biopsies [2], and foetal blood sampling [3]. Many of the minimally invasive procedures are guided by ultrasound (US) imaging as it provides anatomical information at high spatial resolution in real-time, but accurate visualisation of invasive medical devices such as clinical needles can be challenging. During in-plane insertions, thin needles can easily deviate from the narrow US imaging plane, causing loss of visibility. Additionally, needle visualisation is degraded with increasing insertion angles due to specular reflections of US waves from the needle surface. Furthermore, the needle shaft that crosses the imaging plane may be misinterpreted as the tip especially during out-of-plane insertions. Misinterpretation of the needle position, especially the tip position, relative to the patient’s anatomy can cause severe complications [4]. 

Echogenic needles are sometimes used in clinical practice to improve needle visualisation, especially at steep insertion angles during ultrasonography [5]. These needles are made echogenic using different surface designs, but they also produce severe reflection artefacts, and they are not effective with highly echoic background tissue [6,7]. Mechanical tracking devices were also developed for aligning the needle trajectory with the tissue target and the US probe during insertions [8,9,10]. These devices usually require significant systematic calibrations to achieve reasonable tracking accuracy, which increases the complexity of clinical operations. 

Needle tips could be identified using embedded sensors. Electromagnetic (EM) sensors are frequently used for developing computer-assisted procedural applications, but interference sources such as nearby electrical devices and metal subjects can readily perturb their sensitivity [11]. Ultrasonic tracking is a technique that localises the needle tip with integrated US transducers (transmitters or receivers) to enable US communication between the needle tip and the external US imaging probe [12,13]. Xia et al. proposed an ultrasonic tracking system where a fibre-optic US receiver was integrated into a 20 G needle to receive US waves from the probe [14,15,16,17]. As a reciprocal implementation, Mung et al. equipped the catheter tip with a piezoelectric US transmitter [18,19]. US signals transmitted from the tip and received by the probe could be used for estimating the tip location. Fibre-optic US transmitters were also developed for ultrasonic tracking [20]. These miniature transmitters can be readily integrated with invasive devices, such as needles [21,22,23], catheters [24], and guidewires [25], for tip localisation and visualisation. In a recent work by Xia et al. [22], US waves were generated by delivering pulsed light interstitially through an optical fibre integrated within the cannula of a 22 G needle. A nanocomposite absorbing coating was deposited at the distal end of the optical fibre to enhance the US generation. An imaging probe at the tissue surface detected the US emissions to perform needle tip tracking. However, visualisation of the needle shaft was not feasible with that implementation.

Photoacoustic (PA) imaging is an emerging modality that provides rich optical absorption contrast of biological tissue at high spatial resolution and large imaging depths [26,27,28]. With PA imaging, excitation light pulses are absorbed by optical absorbers such as endogenous chromophores in biological tissue, causing rapid and localised increases in temperature. This leads to thermoelastic expansion generating US waves, which can be received by an US probe at the tissue surface. PA imaging can be combined with US imaging to provide complementary information on tissue by sharing a clinical ultrasound imaging probe for US detection. Invasive medical devices such as metallic needles can be better visualised using PA imaging by extracorporeal illumination using light-emitting diodes (LEDs) compared to US imaging due to the high optical absorption of metal [29]. LEDs offer an alternative to conventional solid-state lasers as PA excitation light sources. They could accelerate the clinical translation of PA imaging due to their high affordability, portability, and light safety [30,31]. However, with LED-based PA imaging, the needle visibility is compromised due to the low pulse energy, and unambiguous needle tip detection remains challenging at deep insertion depths. 

Optically absorbing coatings can also be used to enhance the visibility of interventional devices with PA imaging. In Ref. [32], the exterior surface of a 20 G spinal needle and an epidural catheter were coated with a multi-walled carbon nanotube-polydimethylsiloxane (MWCNT-PDMS) nanocomposite. Due to the high optical absorption and PA signal generation efficiency of the MWCNT-PDMS composites, the needle and the catheter could be visualised at depths up to 35 mm with low-fluence-based PA imaging. However, explicit identification of the needle tip remained challenging due to the misinterpretation of the PA signals from the needle shaft.

In this work, we propose a PA/US system that combines both interstitial and extracorporeal illumination to enhance the visualization of the needle tip and shaft during minimally invasive procedures. The candle soot nanoparticle-polydimethylsiloxane (CSNP-PDMS)-coated needle shaft was exposed to the surface illumination provided by LED arrays, and the needle tip was visualised with an embedded optical fibre delivering PA excitation light to the tip coating. The performance was investigated through real-time needle insertions into a biological tissue ex-vivo model. The visibility of the needle with PA imaging, including the needle shaft and the tip, was significantly enhanced by CSNP-PDMS coatings. This method significantly enhanced the visibility of both the needle shaft and tip, especially at steep insertion angles and large depths. 

## 2. Materials and Methods

### 2.1. Photoacoustic-Ultrasound Imaging System with Interstitial and Extracorporeal Illumination 

**Figure 1:**
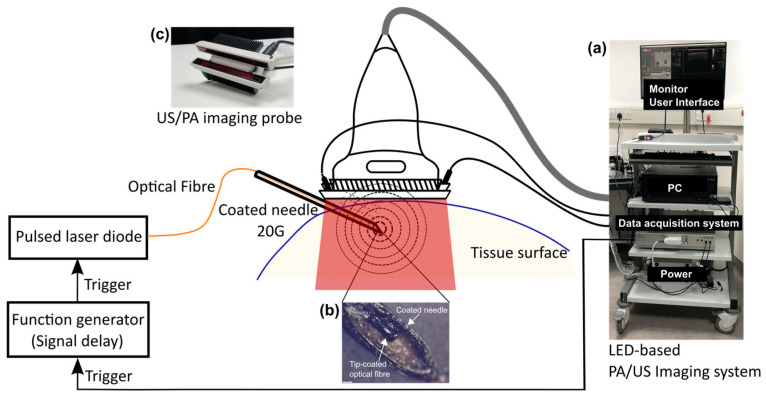
Schematic diagram of the dual-modal photoacoustic (PA) and ultrasound (US) imaging system combining extracorporeal and interstitial illumination with elastomeric nanocomposite coatings for needle enhancement. (**a**) LED-based PA/US imaging system on a trolley; (**b**) Photograph of the needle tip showing a tip-coated fibre inside the lumen providing interstitial illumination for needle tip visualization (scale bar: 200 μm); (**c**) Photograph of the PA/US imaging probe with LED bars providing extracorporeal illumination for needle shaft visualisation.

The system was based on a commercially available LED-based PA/US imaging system (AcousticX, CYBERDYNE INC, Tsukuba, Japan) as shown in Figure 1. It comprised a graphical user interface displayed on the monitor, a PC, data acquisition hardware, and a power supply unit, and was fitted onto a medical trolley (Figure 1a). The imaging probe was constructed by a linear array-based US probe with two LED bars attached at opposite sides to provide extracorporeal illumination.

The fibre-optic US transmitter was made with a CSNP-PDMS composite coating applied onto the distal end of an optical fibre (see Section 2.2). The transmitter was placed into the cannula of a metallic spinal needle (20 G, BD, UK) whose exterior was coated with a CSNP-PDMS composite (shown in Figure 1b). US waves were generated by PA excitation of the coatings both at the fibre end and the needle exterior. The excitation pulses for interstitial illumination were delivered through the optical fibre to the distal end from a laser diode (wavelength: 1550 nm; pulse repetition frequency: 4 kHz, pulse width: 60–150 ns, pulse energy: 300–6000 nJ; L-cube, Laser Components, Chelmsford Essex, UK). The choice of the wavelength for the interstitial illumination in this study is based on the high water absorption at 1550 nm, which allows the generation of strong PA signals at the bare fibre tip.

The signal for externally triggering the laser diode was provided by AcousticX. It was a TTL signal with a default pulse width of 40 μs, but with a 2.12 μs time advance at each pulse compared to the internal triggering for the LED arrays. Therefore, a function generator (Keysight Technologies, Santa Rosa, CA, USA) was used for synchronizing the output before triggering the laser diode with a trigger delay function. The pulsed excitation light from the laser diode was then focused and coupled to the optical fibre. The maximum pulse energy measured at the distal end of the fibre without the coating was 350 nJ. Simultaneously, AcousticX provided pulsed excitation light via two LED bars emitting at 850 nm. Each bar contained four rows of LEDs (36 elements per row; each element is 1 mm × 1 mm). Each bar generated a 200 μJ pulse with a flexible pulse repetition frequency from 1 to 4 kHz and tunable pulse duration from 30 ns to 100 ns. According to [29], a pulse duration of 70 ns was selected for maximising the light pulse energy. With the affixation on the US transducer at a certain declination, the LED bars could illuminate an area of 50 mm × 7 mm at the focal depth of the probe (15 mm) assuming a non-scattering medium with a maximum fluence of 0.11 mJ/cm^2^. 

US signals were received at the tissue surface by an US probe that had a linear transducer array of 128 elements spanning 38.4 mm and a central frequency of 7 MHz. Dual-mode PA/US imaging can be performed in an interleaved manner by sharing the same US probe. Pre-beamformed radiofrequency (RF) data for PA and US imaging acquired from 128 elements were sampled at 20 MHz and 40 MHz simultaneously, followed by image reconstruction using an inbuilt Fourier transform-based reconstruction algorithm [33] on the graphics processing unit (GPU). Therefore, reconstructed PA and US images were displayed on the monitor in real-time. Meanwhile, raw RF data could be saved for offline processing.

### 2.2. Coating Optical Fibres and Medical Needles 

#### 2.2.1. Coating Fabrication 

The fibre-optic transducer was fabricated using a previously developed method [34]. The CSNP-PDMS film was coated onto the cleaved distal end of step-index, silica-core, silica-cladding multimode fibres (NA: 0.22 with core/cladding diameters of 200/240 μm; FG200LCC, Thorlabs, Ely, UK). The fibre end-face was cleaved using an automated fibre cleaver (CT-100, Fujikura, Japan). The PDMS (Sylgard 184, Dow Corning, Midland, MI, USA, Europe) used was prepared by mixing the Sylgard base and curing agent together in a 10:1 volumetric ratio and degassed using a vacuum (3 min).

Briefly, fibres were placed within a paraffin wax candle flame, 2–3 mm above the flame centre, for 3 s to form a CSNP coating at the fibre’s distal end. The fibre was then dip-coated with PDMS and allowed to cure at room temperature in an upright position (48 h). 

Needles were coated with CSNP by slowly translating them within a flame for a period of 5 s to ensure a complete coating of the needle shaft and tip. A PDMS solution was prepared by mechanically mixing a one-part PDMS (MED-1000, Polymer Systems Technology, High Wycombe, UK) with xylene in a 1 g: 1.8 mL ratio. A PDMS overcoat was then applied to the needle using dip-coating methods and the needles were allowed to cure in an upright position at room temperature, for at least 4 h.

#### 2.2.2. Coating Examination 

The CSNP-PDMS coatings deposited onto the fibre end-faces were examined using a stereomicroscope (Leica, London, UK). The texture and surface topology of each film was inspected through end-on illumination, whilst a side-on inspection of the film was used to further examine the coating’s morphology and thickness. To assess the uniformity of the film’s coverage of the fibre end-face, through-film illumination was employed. Besides, the CSNP-PDMS coatings on the needle exterior were delaminated and microscopically inspected. The thickness of the needle coating was estimated using a digital micrometer (Beslands, London, UK). 

#### 2.2.3. Optical Characterisation 

The optical absorption of the CSNP-PDMS fibre-optic films was measured across a wavelength range of 400–1600 nm. Broadband light from a halogen lamp (HL-2000-HP-FHSA, Ocean Optics, Dunedin, FL, USA) was delivered from the proximal end of a fibre and transmitted through the CSNP-PDMS coating at the distal end. Light transmitted through the coating was received in an integrating sphere (FOIS-1, Ocean Optics, USA) and measured with two spectrometers (Flame-T-VIS-NIR, Ocean Optics; 300–1000 nm and NIRQuest512, Ocean Optics, USA; 1000–1800 nm). A non-coated cleaved fibre of the same type was used as a reference and dark measurements were taken to correct for background light.

### 2.3. Imaging with Needle Insertions into Ex Vivo Tissue

PA and US imaging were performed with interstitial and extracorporeal illumination on a pork joint tissue ex-vivo model. Both out-of-plane and in-plane needle insertions were studied. Note that the tough outer tissue was removed before insertions. 

During out-of-plane insertions, a specially designed needle guide was used to help localise the needle tip (see Supplementary Materials; Figure S1). Using the guide under water enabled the inserted needle to follow the desired trajectory where the needle tip showed a good contrast on both the PA and US images. When the tip was in the imaging plane, the insertion length was recorded by labelling the position of the proximal end on the guide. Without moving the needle guide and imaging probe, the pork joint ex-vivo model was used as the tissue target. The needle was re-inserted following the desired trajectory until the proximal end aligned with the previous label, indicating that the tip was in the imaging plane. 

During in-plane insertions, a needle with a tip-coated fibre placed in the lumen and exterior coating was inserted into the tissue at insertion depths spanning from 1 cm to 3 cm and angles ranging from 20 deg to 55 deg. A bare needle with an embedded tip-coated fibre was imaged with the same placements for comparison. In addition, as the optical absorption of water is relatively high at 1550 nm, insertions with a bare needle and the tip-exposed fibre were also performed as a comparison to the tip-coated fibre. 

For quantitative analysis, two performance metrics were considered: the contrast-to-noise ratio (CNR) and the signal-to-noise ratio (SNR). The CNR of the needle tip was calculated for both the PA and US images during out of plane insertions. The CNR was defined as CNR = $\left|\mu_{s}-\mu_{b}\right|\;/\;\sigma_{b}$, where $\mu_{s}$ and $\mu_{b}$ denote the mean of the image amplitude in a signal region and a background region, respectively. The background region was manually selected as a rectangular area close to the signal region. Here, $\sigma_{b}$ is the standard deviation of the image amplitude in the background region.

The SNRs of the needle shaft and the needle tip were calculated at each insertion for quantifying the improvements respectively. The SNR was defined as SNR = S / σ, where S represents the mean of the signal intensity in the region of the needle shaft or tip. The shaft region was annotated as a rectangular box with reference to the real diameter and the visible length on the PA image. The signal region from the needle was labelled as an oval by considering the full width at half maximum (FWHM) values along the x and y axis. Here, σ is the standard deviation of the background region that was extracted by excluding the signal region from the entire image. 

The dataset of each scenario regarding the existence of CSNP-PDMS coatings at the shaft and fibre tip consisted of 10 PA images for SNR measurements. The insertion depths and angles were roughly categorised into three groups; for each insertion depth and angle, images from each scenario were chosen randomly. Paired sample *t*-tests were employed between the bare needle and other scenarios to indicate the significance levels of enhancements.

## 3. Results

### 3.1. Coating Examination

Stereomicroscope images of the coated optical fibres revealed black CSNP-PDMS nanocomposites with a smooth exterior with an even CSNP coating coverage on the fibre end-face and homogenous composition featuring a black colour characteristic of the CSNPs. Through-fibre illumination of the fibres revealed little transmission of the input light characterising the high concentration of CSNPs deposited across the fibre end-face within the PDMS matrix.

**Figure 2:**
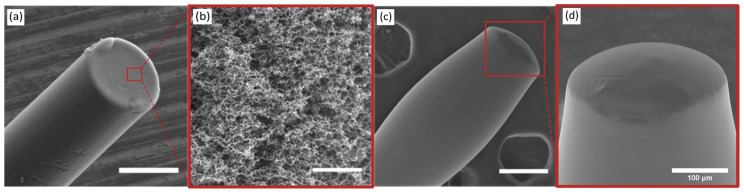
Scanning electron microscopy (SEM) images of the coating on the end face of the optical fibre where (**a**) is the CSNP coating, (**b**) is a magnified view of the CSNP nanostructure, (**c**) is the CSNP-PDMS bilayer nanocomposite coating, and (**d**) is a magnified view of the CSNP-PDMS coating. Scale bars are 200 μm for (**a**,**c**), 5 μm for (**b**), and 100 μm for (**d**).

SEM images indicated that the CSNP coating on the distal end of the fibre presented a convex shape with good coverage of the fibre end-face (Figure 2a). SEM imaging also showed that the CSNP fibre coating had a mesh-like structure (Figure 2b), and the PDMS overcoat obscured this microstructure, presenting a smooth, domed surface (Figure 2c). The thickness of the CSNP-PDMS coating of a sample needle was around 53 μm.

### 3.2. Optical Characterisation 

**Figure 3:**
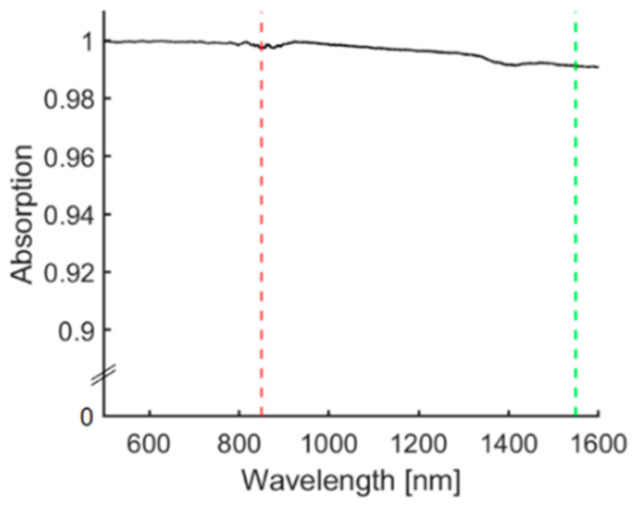
Absorption spectrum of CSNP-PDMS nanocomposite deposited onto the distal end of an optical fibre with absorption highlighted at 850 nm (dashed red line) used for PA generation of needle exterior and 1550 nm (dashed green line) used for optical ultrasound generation of the fibre tip.

The optical absorption spectrum of the CSNP coating is shown in Figure 3. The film had a high, broadband optical absorption at both wavelengths for optical US generation from the fibre-optic film (1550 nm) and PA excitation (850 nm), measuring >99% absorption across the wavelength range of 400–1600 nm. 

### 3.3. Out-of-Plane Insertions

**Figure 4:**
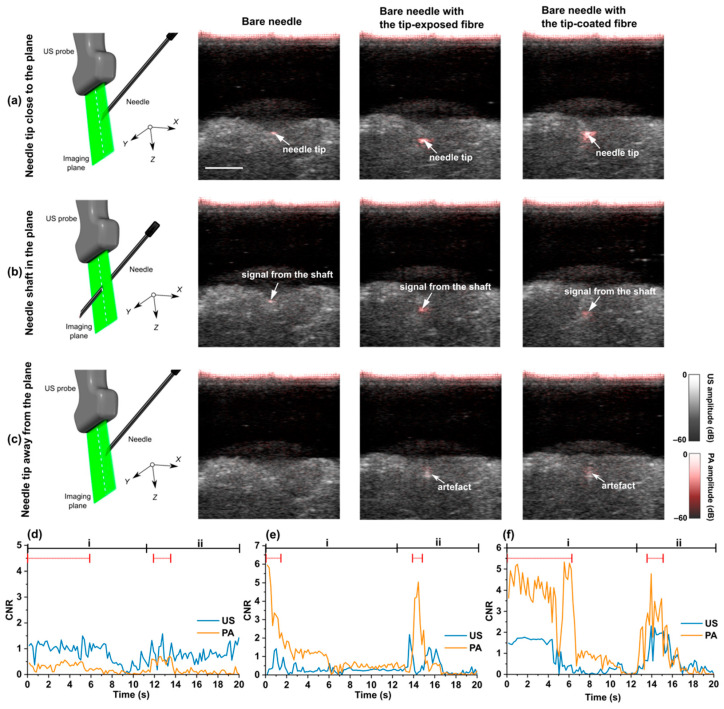
Overlaid photoacoustic (PA) and Ultrasound (US) images of out-of-plane insertions into pork joint ex-vivo using a bare needle, a bare needle with a tip-exposed fibre, and a bare needle with a tip-coated fibre. (**a**–**c**) schematic illustrations of the needle tip location during out-of-plane needle insertions: (**a**) needle tip close to the imaging plane, (**b**) needle shaft in the imaging plane, and **(c)** needle tip away from the imaging plane. (**d**–**f**) show the contrast-to-noise ratios (CNRs) of the needle in PA and US images during a 20 s out-of-plane procedure; i: needle was inserted towards the imaging plane; ii: needle was withdrawn from the imaging plane; time intervals covered by the red dotted lines correspond to the needle tip in the imaging plane (approximately 0–6.1 s and 12.2–13.7 s in (**d**); 0–1.8 s and 13.6–14.9 s in (**e**); 0–6.5 s and 13.3–15.7 s in (**f**)); Images were obtained from real-time reconstruction and displayed on a logarithmic scale. All the images have the same scale bar of 1 cm.

Figure 4 shows three representative results of the out-of-plane insertions where the needle was inserted from outside the imaging plane. For a bare needle, as shown in Figure 4a,b, the intersection between the needle shaft and the imaging plane presented similar visual features to those of the needle tip within the imaging plane. A tip-exposed (uncoated) fibre placed inside the bare needle improved the visualisation of the needle tip due to water light absorption in front of the fibre end within the tissue (Figure 4a; Bare needle with the tip-exposed fibre), making it possible to identify the needle tip from the needle shaft. Robust enhancement on the needle tip could be observed using a bare needle but with a tip-coated fibre (Figure 4; Bare needle with the tip-coated fibre). Compared to the bare needle with or without the tip-exposed fibre, artefacts from the needle shaft were less visible (Figure 4b; Bare needle with the tip-coated fibre). It is noted that a CSNP-PDMS nanocomposite coating on the distal face of the fibre resulted in significant enhancements around the tip, which could even be visualised when the needle was being withdrawn from the imaging plane (Figure 4c; Bare needle with the tip-coated fibre). Videos obtained in real time were provided (Supplementary Materials; Videos S1–S3), where out of plane insertions were carried with the bare needle, the bare needle with the tip-exposed fibre, and the bare needle with the tip-coated fibre. 

The visual improvement of the needle tip identification was quantified by measuring CNR in PA and corresponding US images and shown in Figure 4d–f. With the tip-coated or exposed optical fibre inside the needle lumen (Figure 4e,f), the CNRs of the tip in PA images were higher than those in the US images when the needle tip was in the imaging plane (denoted by red dotted lines). Furthermore, the CNR for PA images decreased when the needle shaft was in the imaging plane, or the needle was withdrawn from the imaging plane. This indicated that discrimination between the needle shaft and tip could be achieved by using the optical fibre (tip-coated or tip-exposed) and interstitial illumination with PA imaging. 

### 3.4. In-Plane Insertions 

**Figure 5:**
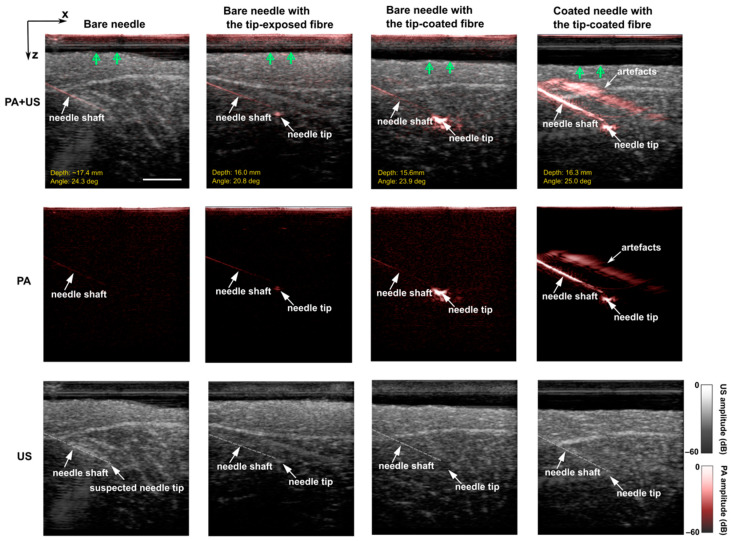
Overlaid Photoacoustic (PA) and Ultrasound (US) images, PA images, and US images acquired during in-plane needle insertions into a pork joint tissue ex-vivo with a bare needle, a bare needle with a tip-exposed fibre, a bare needle with a tip-coated fibre, and a coated needle with a tip-coated fibre. Insertion depth was defined as the shortest distance from the suspected needle tip to the skin surface (denoted by green arrows). Insertion angles were measured from the positive x-axis going clockwise. In US images, needles including the tip were marked (translucent white dashed lines) according to corresponding PA images. Images were obtained from real-time reconstructions and displayed on a logarithmic scale. All the images have the same scale bar of 1 cm.

For in-plane insertions, PA imaging could render better visualisation of a bare needle than US imaging but suffered from low SNRs (Figure 5; Bare needle). In Figure 5, three enhancement cases were validated and compared with the bare needle regarding the enhancements on both the needle shaft and needle tip. For each case, one representative procedure with a clinically relevant insertion depth and angle was chosen. Additional results with a wide range of insertion depths and angles (Figure S2) and videos (Videos S4–S7) are included in Supplemental Materials. 

Due to the shallow depth and small insertion angle, the bare needle was visible at the proximal section close to the tissue surface but was barely visible toward the needle tip. Moreover, for in-vivo scenarios, background signals from blood vessels would further affect the needle visibility, especially the needle tip. The needle tip was visualised with PA imaging by integrating the tip-exposed fibre into the bare needle (Figure 5; Bare needle with the tip-exposed fibre). Light delivered through the optical fibre was absorbed by the radiated medium in front of the exposed tip due to high optical absorption of water at 1550 nm, providing the PA contrast at the tip area. However, the needle tip visibility in PA images decreased significantly at larger depths (see Supplementary Materials; Figure S2). This could be compensated by applying a CSNP-PDMS coating on the fibre distal face. Coating onto the fibre end generated substantial visual enhancement on the needle tip, demonstrating a 5.5- and 3.7-fold improvement in SNR of the tip compared to the bare needle (*p* < 0.0001) and the tip-exposed fibre (*p* < 0.001), respectively. It is noticeable that the strong PA signal generated from the tip-coating overwhelmed the contrast of the needle shaft, especially for the distal segment (Figure 5; Bare needle with the tip-coated fibre). As expected, coating both the needle shaft and the fibre tip rendered better visibility compared to those without coatings, as shown in the last column of Figure 5.

**Figure 6:**
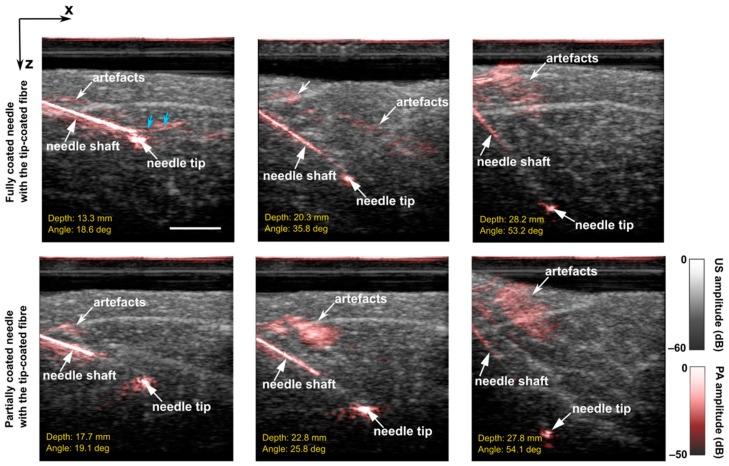
Overlaid Photoacoustic (PA) and Ultrasound (US) images acquired during in-plane needle insertions into a pork joint tissue ex vivo with a fully coated needle and a partially coated needle (tip-coated fibres inside the lumens). Blue arrows denote arc-shaped artefacts from the needle tip. Images were obtained from real-time reconstructions and displayed on a logarithmic scale. All the images have the same scale bar of 1 cm.

To facilitate the separation of the PA signals from the needle shaft and tip, two different coating strategies were compared. First, the entire needle shaft was coated (around 3.81 cm), and second, the needle shaft was coated from the proximal end until ca. 1 cm measured from the needle tip, leaving an uncoated region. Figure 6 compares their performance during in-plane insertions into pork joint tissue ex vivo. When the insertion angles were smaller than 20 deg (shown in the first column), arc-shaped artefacts (blue arrows) that originated from the needle tip became visible when the needle was fully coated. These artefacts overlapped with the signal generated from the needle tip. This problem was mitigated by using the partially coated needle. Further comparisons were performed with large insertion angles and depths (shown in the second and third columns). It is worth noting that even with the full-length needle coating, the distal segment of the needle shaft was barely visible at large depths due to the massive light attenuation in tissue with the surface illumination.

### 3.5. SNR Analysis 

**Figure 7:**
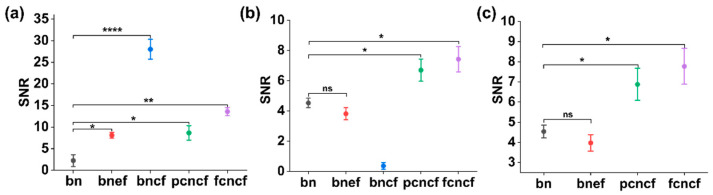
Signal-to-noise ratio (SNR) measurements for photoacoustic images acquired during in-plane needle insertions into a pork joint tissue ex vivo. (**a**) SNR improvement on the needle tip, (**b**) SNR improvement on the needle shaft, and (**c**) SNR improvement on the needle including the needle shaft and tip. bn: bare needle; bnef: bare needle with the tip-exposed fibre; bncf: bare needle with the tip-coated fibre; pcncf: partially coated needle with the tip-coated fibre; fcncf: fully coated needle with the tip-coated fibre. Data represent mean values and error bars represent standard errors. * *p* < 0.05, ** *p* < 0.01, **** *p* < 0.0001, ns: not significant.

The SNR was separately measured at the needle tip and shaft using PA images and shown in Figure 7a,b respectively. The improvements of SNR on both the needle tip and shaft were shown in Figure 7c. The performance of the bare needle with the tip-coated fibre was biased towards the needle tip enhancement, and thus was not considered in Figure 7c. A paired samples t-test was performed to compare the statistical difference of the SNR measurements with the bare needle and coatings applied on the fibre tip or needle shaft. In Figure 7a, compared to the bare needle, the tip visibility was significantly enhanced by embedding the optical fibre with or without the coating (*p* < 0.05 for the bare needle with the bare fibre and the partially coated needle with the tip-coated fibre; *p* < 0.01 for the coated needle with the tip-coated fibre). It is noted that the most significant improvement of the tip visibility was achieved by only employing the coating on the fibre tip (*p* < 0.0001). In contrast, the assembly using the tip-exposed fibre and the bare needle did not benefit the shaft visualisation and the comprehensive enhancement, as shown in Figure 7b,c. Two different coating strategies on the needle exterior demonstrated slight differences in SNR. It is reasonable that coating the proximal end of the needle rather than the full length brought less enhancement on the shaft (shown in Figure 7b, the SNR was 6.87 ± 1.77 for the partially coated needle and 7.77 ± 1.98 for the fully coated needle, respectively).

## 4. Discussion

Clear visualisation of medical devices such as metallic needles is essential in minimally invasive procedures, where it is particularly useful for guiding the needle towards the procedure target and avoiding damaging critical tissue structures, thus improving the efficacy and safety of the procedures. Needle visibility in US images can be poor due to specular reflections, and misinterpretation between the needle shaft and tip can cause severe complications during out-of-plane needle insertions. PA imaging has shown promise for imaging clinical needles, even with low-fluence light sources such as LEDs. However, the output energy of LEDs is much lower than those of solid-state lasers, leading to suboptimal imaging quality manifested in reduced SNRs and CNRs. In addition, the maximum imaging depth with the LED-based extracorporeal illumination is around 2 cm in vivo, which is insufficient for clinical applications with deep procedure targets such as fetal interventions. In this work, we achieved robust enhancements in the visualisation of both the needle shaft and tip with PA imaging, by combining extracorporeal and interstitial illumination with nanocomposite coatings. The CSNP-PDMS composites, enabling high optical absorption and signal generation efficiency in PA imaging, were proposed for coating the fibre tip embedded in the needle lumen and the needle exterior. The CSNP-PDMS composites adhere sufficiently to the fibre-optic distal end and needle exterior, which enable the devices to be repeatedly inserted into the pork joint ex-vivo tissue. These composites have been produced using an inexpensive and facile fabrication method and produced comparable generated ultrasound measurements across a variety of fabricated composites, which have led to similar enhancements in contrast in inserted needles. 

The performance was validated by comparing a coated needle containing an optical fibre (tip-exposed or tip-coated respectively) with a bare needle during out-of-plane and in-plane procedures ex vivo. During out-of-plane insertions, the visualisation of the needle tip was enhanced by the integrated optical fibre (tip-exposed or tip-coated) and interstitial illumination, which could be helpful for visually discriminating the needle shaft and tip with PA imaging. With the fibre tip uncoated, PA signals were generated from the medium in front of the fibre due to the strong optical absorption of water at 1550 nm. Coating applied onto the fibre tip further increased the contrast of the needle tip, and more importantly, the strength of the generated PA signal is not affected by the medium in front of the fibre-tip because of the high US generation efficiency of the coating (Figure 3). Additionally, the results from the in-plane insertions show that the composites’ optical absorptions were sufficient for visually enhancing both the needle shaft and tip in PA imaging with the illuminations from the tissue surface and delivered to the fibre end respectively. The exterior coating on the needle shaft enhanced the shaft visualisation at different insertion angles and depths with the extracorporeal illumination of the LED arrays. Instead of coating the entire needle, coating restricted to the proximal segment of the shaft was investigated. Experimental results showed that keeping the distal area of the needle uncoated could avoid the interference that stemmed from signals from the shaft by the full-length coating, and therefore, enabled the ambiguous identification of the needle tip. Noticeably, the needle could be visualised at depths down to 38 mm (maximum displaying depth of AcousticX). The fully coated needle with the tip-coated fibre embedded improved the overall SNR by a factor of 1.7 compared to the bare needle. However, it should be noted that the coatings also strengthened the artefacts derived from the needles. Those above the needles were highly likely associated with the limited aperture of the imaging probe and the side lobes of ultrasound beams. Other artefacts appearing as multiple parallel lines to the needle shaft could stem from the ultrasound reverberation within the needle lumen. Those artefacts could be removed by a deep learning-based framework developed for improving the needle visualisation with PA imaging as demonstrated in a previous study [35]. 

Optical ultrasound transducers can efficiently generate high-amplitude ultrasound via the PA effect with optically absorbing elastomeric composites [36,37,38]. CSNP-PDMS composites feature highly optically absorbing CSNPs embedded within the transparent PDMS elastomer, which possesses a large volumetric thermal expansion coefficient [39]. CSNP-PDMS composites have already shown great promise when deposited onto planar [40] and convex [41] macroscale surfaces, where the latter has demonstrated High Intensity Focussed Ultrasound (HIFU), a therapeutic technique used for drug delivery [42], the ablation of tumours [43], and bones [44]. Curved CSNP-PDMS composites employed for HIFU have generated pressures above 20 MPa, with bandwidths in excess of 20 MHz. Owing to the highly hierarchical structure of the CSNPs, CSNP-PDMS composites exhibit high PA conversion efficiencies that compare well with other materials used for optical ultrasound generation such as carbon nanotubes [34,45]. Little research has investigated the application of CSNP-PDMS nanocomposites onto the distal ends of optical fibers, forming miniaturised optical US transmitters [39].

To assess the suitability of CSNP-PDMS composites in needle tracking during surgical interventions, a detailed characterisation of the mechanical and chemical properties, and biocompatibility of these composites should be undertaken. There should be further evaluation of the properties of CSNPs produced by using different candle waxes, wicks, and collection distances above the candle flame to examine their effects, if any, on the resulting composite [46]. The composite coated on the needle exterior is exposed directly to the imaged tissue during insertions and thus experiences a higher degree of abrasion from the tissue. For this reason, this composite must be more robust than the composite deposited on the fibre distal end inserted within the needle. The fibre-optic composite also has further protection from the tissue since it is retracted from the end of the needle and secured firmly with an adhesive. The application of a further polymer overcoat to the CSNP-PDMS composite on the needle’s exterior should provide this required increase in resistance to abrasion. Moreover, the PDMS overlayer should be able to infiltrate the highly arborised structure of CSNPs, to a large extent enabling its strong adhesion to the underlying substrate, which contributes to the composite’s robustness. In addition, when under sustained illumination, both composites must be resistant to photobleaching, which would cause the generation of US to deteriorate. In the future, the variations of the thicknesses of the coatings between different needles and fibres could be statistically investigated. The repeatability of different fabrication methods could also be compared.

Another aspect of consideration is the extent of leaching of the CSNPs from the PDMS when these composites are used for extended periods of time as required during surgical interventions. CSNPs are not anticipated to leach from a PDMS overcoat with a minimum thickness of 40 microns (see Figure 2d). The concern of leaching is of lower risk than the composite being dislodged by abrasion. Some studies measured the effects of exposure of CSNPs to non-human species and concluded that moderate exposure to CSNPs can provoke cellular mechanisms that lead to cytotoxicity [47,48]. However, no overall changes to the species’ organs were observed. However, exposure to CSNPs is commonplace as shown by the availability of candles for personal use, and their incorporation within the human body has not yet been documented or studied. Furthermore, the PDMS matrix that encompasses the CSNPs is a certified medical-grade material, removing any concerns over its interaction with tissue. The issue of toxicity would, like mechanical stability, be more critical for the composite on the needle’s exterior rather than the fibre-optic coating, given the former’s proximity to tissue.

## 5. Conclusions

We propose a method to enhance needle visualisation in PA imaging by combing interstitial and extracorporeal illumination with CSNP-PDMS nanocomposite coatings. We demonstrate that the needle shaft and tip could be clearly visualised ex vivo in deep tissue with significant improvements achieved by the highly absorbing coatings during out-of-plane and in-plane insertions. We conclude that the proposed method could be useful in various minimally invasive procedures by providing an accurate visualisation of interventional medical devices.

## Data Availability

The data that support the findings of this study are available on request from the corresponding authors, S.N. and W.X.

## Supplementary Materials

The following supporting information can be downloaded at: https://www.mdpi.com/article/10.3390/s22176417/s1, Figure S1: Photograph (a) and schematic diagram (b) of out-of-plane insertions with a needle guide; Figure S2: Overlaid photoacoustic and ultrasound images acquired during in-plane needle insertions into a pork joint tissue ex vivo with a bare needle, a bare needle with a tip-exposed fibre, a bare needle with a tip-coated fibre, and a coated needle with a tip-coated fibre; Videos S1–S3: Real-time photoacoustic and ultrasound visualisation of a bare needle (S1), a bare needle with a tip-exposed fibre (S2), and a bare needle with a tip-coated fibre (S3) during out-of-plane insertions into a pork joint ex-vivo model; Videos S4–S7: Real-time photoacoustic and ultrasound visualisation of a bare needle (S4), a bare needle with a tip-exposed fibre (S5), a fully coated needle with a tip-coated fibre (S6), and a partially coated needle with a tip-coated fibre (S7).
