# Supplementary material for: Enhanced Photoacoustic Visualisation of Clinical Needles by Combining Interstitial and Extracorporeal Illumination of Elastomeric Nanocomposite Coatings
Source: Sensors (Basel). 2022 Aug 25;22(17):6417. doi: 10.3390/s22176417 (PMC9460224; doi:10.3390/s22176417)
Supplement: Supplementary file 1 [file sensors-22-06417-s001.zip › sensors-1848840-supplementary.pdf]

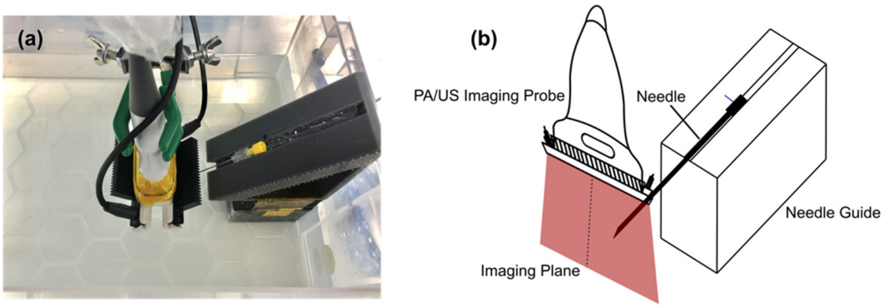

Figure S1. Photograph **(a)** and schematic diagram **(b)** of out-of-plane insertions with a needle guide.

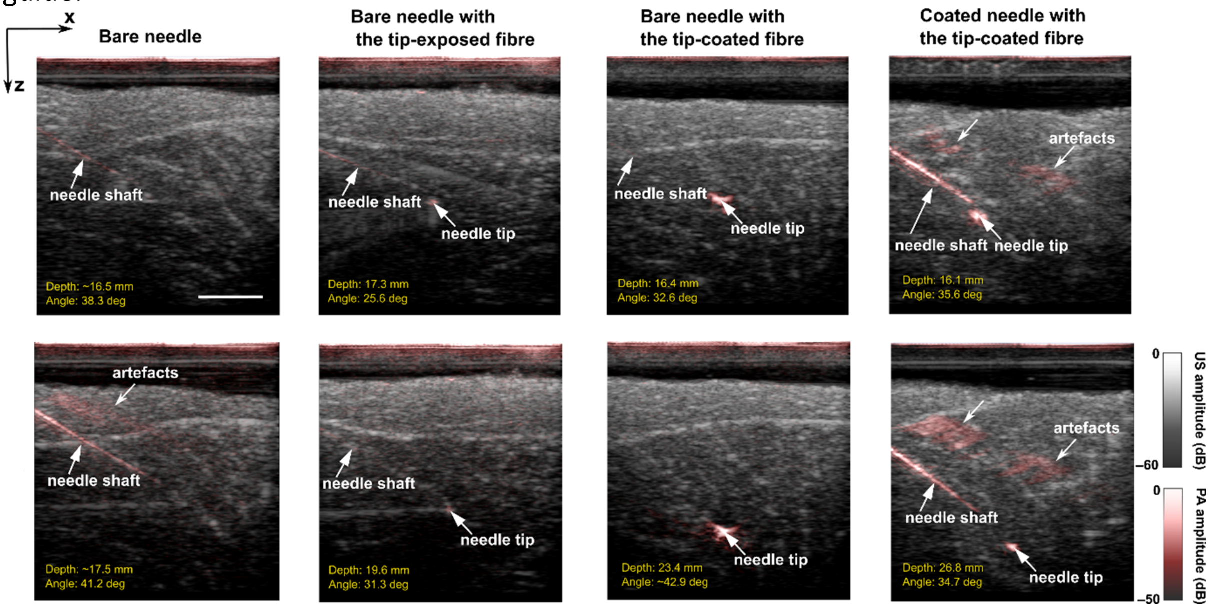

Figure S2. Overlaid photoacoustic and ultrasound images acquired during in-plane needle insertions into a pork joint tissue ex vivo with a bare needle, a bare needle with a tip-exposed fibre, a bare needle with a tip-coated fibre, and a coated needle with a tip-coated fibre. Images were obtained from real-time reconstructions and displayed on a logarithmic scale. All the images have the same scale bar of 1 cm.
